# Key components of mechanical work predict outcomes in robotic stroke therapy

**DOI:** 10.1186/s12984-020-00672-8

**Published:** 2020-04-21

**Authors:** Zachary A. Wright, Yazan A. Majeed, James L. Patton, Felix C. Huang

**Affiliations:** 1 Department of Bioengineering, University of Illinois at Chicago, Chicago, IL USA; 2 Arms + Hands Lab, Shirley Ryan AbilityLab, Chicago, IL USA; 3 Department of Mechanical Engineering, Tufts University, Medford, MA USA

**Keywords:** Stroke, Robotic therapy, Upper limb, Energetics, Neurorehabilitation, Outcomes

## Abstract

**Background:**

Clinical practice typically emphasizes active involvement during therapy. However, traditional approaches can offer only general guidance on the form of involvement that would be most helpful to recovery. Beyond assisting movement, robots allow comprehensive methods for measuring practice behaviors, including the energetic input of the learner. Using data from our previous study of robot-assisted therapy, we examined how separate components of mechanical work contribute to predicting training outcomes.

**Methods:**

Stroke survivors (*n* = 11) completed six sessions in two-weeks of upper extremity motor exploration (self-directed movement practice) training with customized forces, while a control group (*n* = 11) trained without assistance. We employed multiple regression analysis to predict patient outcomes with computed mechanical work as independent variables, including separate features for elbow versus shoulder joints, positive (concentric) and negative (eccentric), flexion and extension.

**Results:**

Our analysis showed that increases in total mechanical work during therapy were positively correlated with our final outcome metric, velocity range. Further analysis revealed that greater amounts of negative work at the shoulder and positive work at the elbow as the most important predictors of recovery (using cross-validated regression, R^2^ = 52%). However, the work features were likely mutually correlated, suggesting a prediction model that first removed shared variance (using PCA, R^2^ = 65–85%).

**Conclusions:**

These results support robotic training for stroke survivors that increases energetic activity in eccentric shoulder and concentric elbow actions.

**Trial registration:**

ClinicalTrials.gov, Identifier: NCT02570256. Registered 7 October 2015 – Retrospectively registered,

## Background

Assistance is often provided to aid limb movement during the rehabilitation process of stroke survivors. Many clinical researchers agree that active participation enhances recovery, and the goal of therapy should be to maximize “involvement” [1, 2]. Too much assistance can actually discourage patient effort [3]. However, measurement of the degree to which patients are *actually* active is often difficult. Advances in rehabilitation devices allow for the measurement of forces *and* motion to better monitor patient activity. Here we investigate how upper limb mechanics during training relate to recovery.

Current tools for measuring physical activity during therapy offer limited information for describing interaction with the external environment or agent. While studies have shown that the intensity of therapy influences patient improvement, researchers have relied on simple metrics related to experimental conditions (e.g. movement repetitions, time-on-task, and therapy dosage) [4, 5]. More sophisticated tools have been used to directly measure energetic contributions during therapy, such as oxygen consumption devices to measure metabolic cost [6] or electromyography to measure muscle activity [7, 8]. However, such measures do not account for the time-varying force-motion relationships that occur during assisted movement. Robots easily measure both kinematic and kinetic variables facilitating the computation of energetic contributions in terms of mechanical power and work.

While energetic descriptions of movement have been widely studied, it has mainly focused on cyclic [9] or sustained movements, such as walking. Researchers have computed work and power to characterize normal and abnormal gait patterns [10, 11], to evaluate robot-assisted locomotion [12], and to reduce energetic costs when using exoskeletons [13]. Recently our work has focused on robotic augmentation of upper limb dynamics to facilitate vigorous movement during practice [14, 15]. We showed that stroke survivors increase total work output during force training [16]. Our intervention was fundamentally different than many previous strategies in that patients trained over a broader range of movements. In contrast to reaching studies [17, 18], such self-directed exploration allows for the examination of how energetics might depend on different force and motion states.

To better evaluate the variation in patient energetics, we believe more comprehensive measures are required beyond total expenditure of power or work. Researchers have also examined compartmentalized work and power measures in normal limb behaviors, for example, associating magnitudes of mechanical energy (e.g. positive/concentric and negative/eccentric work) with movement actions (e.g. flexion and extension) at individual joints [19]. Motor impairments due to stroke are also typically described in the context of motor actions of the limb. For example, stroke survivors exhibit abnormal flexion and extension synergies [20] and alterations in concentric and eccentric muscle contractions [21, 22]. As such, impairments can be associated with *subcomponents* of work and power. As patients interact differently in response to forces, subcomponents of work and power could reveal individual differences in involvement.

An emerging trend in rehabilitation is to identify certain factors that predict individual improvement in response to therapy. Researchers have identified patient biomarkers (impairment level, neurophysiological) correlated to patient outcomes providing better recommendations for therapy [23–25]. Similarly, our goal is to determine if particular types of work are more important to patient recovery. Such evaluation could inform decisions on design strategies and optimize assistance to each individual. In contrast to previous studies which have relied on independent analyses of many individual predictors, our analysis goal necessitates more rigorous statistical methods to deal with potentially related work features. One possible solution is to employ multiple regression analysis which can identify features most important for prediction.

In this paper, we investigate how the energetic contributions of stroke survivors during robot-assisted training relate to upper limb recovery. We employ well-established methods of inverse dynamics to estimate the torques generated by each patient during self-directed motor exploration training with customized forces. These methods conveniently allow us to quantify the energetic involvement of each individual joint in terms of mechanical work. We then use multiple regression analysis to identify which components of work are most important for predicting recovery. We hypothesize that positive work (concentric) in elbow extension is the best predictor of outcome. This study provides a key preliminary step towards evaluating energetic descriptions of patient involvement which can inform methods for upper limb robotic therapy practice.

## Methods

### Study participants

This investigation considered data collected from a previous study that featured 22 stroke survivors [15]. The main inclusion criteria included: 1) chronic stroke (8+ months post-stroke) 2) hemiparesis with moderate to severe arm impairment measured by the upper extremity portion of the Fugl-Meyer Assessment (UEFM score of 15–50) 3) primary cortex involvement. Each individual gave informed consent in accordance with the Northwestern University Institutional Review Board (IRB).

### Apparatus

**Fig. 1:**
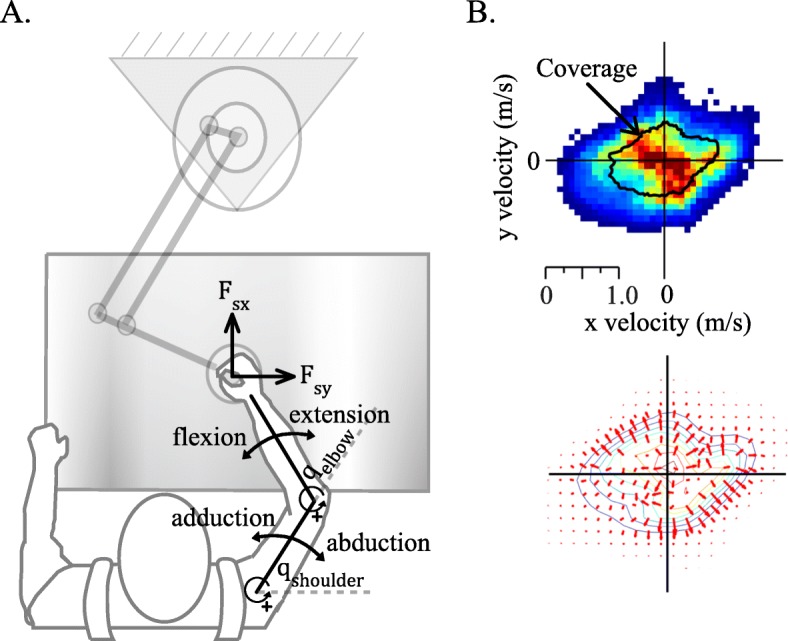
Experimental design. **a** Stroke survivors performed self-directed motor exploration by moving the robot handle in the horizontal plane. Measurements of their limb motion and the interaction forces were used to estimate the positive (concentric) and negative (eccentric) mechanical work exerted in different directions of shoulder and elbow joint motion. **b** The probability distribution of each individual's movement velocities during unassisted motor exploration (top; blue indicates lower probability, red indicates higher probability, black contour line represents the 90th percentile velocity coverage) formed the basis for the design of customized training forces (bottom; red arrows indicate the direction and relative magnitude of forces applied, colored contour lines represents Gaussian model fit to velocity data)

Experiment participants were asked to operate a two-degree of freedom robotic device with the affected arm (Fig. 1a). A custom video display system (not shown) provided visual feedback of the location of the wrist as the arm moved in the horizontal plane. During movement, the weight of the arm was supported. Movement data was collected at 200 Hz and filtered using a 5th order Butterworth low pass filter with a 12 Hz cutoff. Using anthropometric measurements recorded from each participant, we computed inverse kinematic relationships to obtain elbow and shoulder joint angles corresponding to endpoint position data. The robot control and instrumentation were mediated by a Simulink-based XPC Target computer, with a basic rate of 1 kHz. The robot controller compensated for the dynamics of the robot arm. A force sensor attached to the end-effector measured the human-robot interaction forces.

### Experimental protocol

Each participant completed nine sessions across 5 weeks, including evaluation (Baseline, sessions 1, 2; Post, sessions 8, 9) and training (sessions 2–7, spanning 2 weeks) sessions. For each evaluation, participants completed a clinical assessment and a motor performance assessment which included three motor tasks using the robotic device under no forces: point-to-point reaching, circular movements, and six two-minute trials (12 min in total) of self-directed motor exploration. For each training session, each participant first completed a performance assessment then completed an additional 16 two-minute trials (32 min in total) of motor exploration, either in the presence of a customized force field (Force group, *n* = 11) or absent forces (Control group, *n* = 11). This investigation considered only data from the motor exploration portions of Baseline (session 2) and Post (session 8) evaluation sessions as well as the training sessions.

For the motor exploration task, participants were instructed to move the robot handle to all reachable points within a 0.6 m × 0.4 m workspace, to vary their speed and direction of movement as much as possible and to avoid repetitive movements. Each motor exploration trial ended after two cumulative minutes of movement within the workspace. Movement speed below 0.04 m/s was considered rest so that the time samples did not count towards the total movement time. While we informed participants they could rest at any time, we also provided designated rest periods (1–3 min) at the end of the motor performance assessment and prior to the start of training and after the first eight trials during training. After each trial of motor exploration, we provided a *Post-Trial feedback score* summarizing their motor exploration performance, as described previously [15]. The score was based on a heuristic measure of randomness which was used to encourage more variety in movement patterns. Following each completed trial, we displayed on the screen both the “Current” score (score from the most recent trial) and the “Best” score (highest score across all trials) the participant achieved within a given session.

### Design of customized force field

The motor exploration portion of the performance assessment for each training session served as a basis for the design of a customized force field for training in that session (See Fig. 1). To serve as a model of an individual’s typical patterns of movement, this study focused on velocity data accumulated across 12 min of motor exploration. We fit this data with a multivariate Gaussian smoothing function. The result of this model fitting procedure can be visualized by constructing a two-dimensional probability distribution representing the most and least typical movement velocities during exploration (Fig. 1b, colored contours). Next, computing the gradient of the analytical form of this function results in a continuous velocity-dependent function whose output are vectors that represent the slope along the two-dimensional distribution. In principal, the direction of the vectors point from higher probabilities towards lower probabilities of the distribution (Fig. 1b, red arrows). The vector field represents the direction and relative magnitude of robot-applied forces which were updated continuously based on the current velocity of its endpoint. Additional details on the experimental procedures were recently published [15].

### Model of upper limb dynamics

Here we describe the human-robot dynamic interaction for two degree of freedom planar movement (Fig. 1a). We employed established methods of inverse dynamics of upper limb motion to estimate the elbow and shoulder joint torques generated by the human. This analysis considers the human arm as a closed system where an external force at the wrist is available from the force sensor measurements. Thus, the model considers the influence of the torques acting on each joint; including, the torque required to move the arm passively (*τ*_*p*_) which is composed of the torque generated by the human (*τ*_*h*_) and the torque acting on the arm by the robot (*τ*_*r*_). The passive load of the arm can be expressed as ${\tau}_p=M(q)\ddot{q}+C\left(q,\dot{q}\right)\dot{q}$, where *q* represents the joint angles of the arm, *M* is the inertial matrix function and *C* is the Coriolis-centrifugal matrix function. Anatomical measurements of limb segments, body weight and height for each patient were used to estimate the mass distribution of the arm [26]. We computed the torques of the robot acting on the human arm arising from the robot contact forces according to ${\tau}_r={J}_h^T{F}_s$, where *J*_*h*_ is the Jacobian matrix of the arm and *F*_*s*_ is the interaction force measured from the force sensor.

### Model features

We constructed a set of candidate model features (nine in total) to be used as our model predictors in our regression analysis. These features included a single categorical factor representing training group in addition to eight individual data variables, specifically the components of mechanical work relating to each patient’s overall energetic contribution to limb motion during training (Table 1). To compute the work features, we first solved for the patient-generated torque at each individual joint and then calculated the mechanical power $\left(P(t)={\tau}_h\dot{q}\right)$ for each two-minute trial of motor exploration within training (96 trials in total across six training sessions). In principal, the integral of power across time represents the total mechanical work (*W* =  ∫ *P*(*t*)*dt*). We divided the time-series calculations of power into four separate components for each joint. Each of these components represented a different combination of the direction of joint torques generated by experiment participants and the relative direction of angular motion at each respective joint. Finally, we computed the numerical integral for each time series of power to obtain work features; including, both the positive (concentric) and negative (eccentric) work performed in elbow flexion and extension and in shoulder *horizontal* adduction and abduction (Fig. 1a). We represent each individual feature of work as the average across training trials subtracted by the respective average work across unassisted motor exploration trials (six) during Baseline evaluation (session 2).

**Table 1:** Table 1: Individual Participant Data

	Outcomes^a^		Work Features^b^							
	UEFM	Velocity Coverage (m^2^/s^2^)	(−) Shoulder Adduction	(+) Shoulder Adduction	(−) Shoulder Abduction	(+) Shoulder Abduction	(−) Elbow Flexion	(+) Elbow Flexion	(−) Elbow Extension	(+) Elbow Extension
**Force Group**										
1	0.5	1.44	200.9	148.8	251.7	126.6	143.1	86.8	148.4	53.5
2	2	1.36	179.7	58.2	258.0	77.5	179.6	80.4	161.7	37.6
3	2	0.65	51.6	7.9	53.4	13.7	39.2	4.7	30.0	10.1
4	1.5	0.51	119.0	49.9	155.1	46.3	70.9	14.6	83.3	12.9
5	0.5	1.07	108.7	− 40.9	134.4	−1.3	288.9	107.7	251.6	106.7
6	0.5	0.15	46.6	13.7	49.7	22.3	59.1	13.2	59.1	10.2
7	0	2.34	144.8	52.0	173.1	60.6	110.5	54.5	92.2	45.6
8	3.5	0.15	18.5	−7.2	26.2	−0.2	83.6	9.4	61.3	10.5
9	−1	0.67	87.2	22.8	92.8	22.7	48.2	16.0	40.7	13.4
10	2.5	0.25	98.2	26.6	107.7	44.7	69.3	20.4	54.7	17.8
11	0.5	1.14	157.0	82.5	189.7	64.8	152.9	48.8	155.5	39.7
Mean	1.1	0.88	110.2	37.7	135.6	43.4	113.2	41.5	103.5	32.5
± SD	1.3	0.67	57.5	50.1	78.7	38.1	74.1	36.3	67.9	29.4
**Control Group**										
1	0	−0.07	15.5	24.5	17.0	11.8	17.3	26.9	33.5	15.3
2	2	0.01	15.4	6.5	18.9	18.7	15.7	16.4	12.7	20.3
3	2	0.35	−9.0	−15.6	−0.4	5.0	−1.5	18.6	−36.5	31.0
4	3	0.70	14.1	23.0	11.8	23.4	14.9	−1.1	7.2	3.8
5	2.5	1.56	16.6	27.3	23.8	26.8	8.0	2.0	4.6	4.8
6	0.5	0.84	13.7	10.4	11.0	30.3	5.1	−2.0	2.9	2.7
7	2	1.03	15.1	15.1	8.3	18.3	4.8	4.1	7.4	6.5
8	−0.5	1.28	11.5	10.5	13.2	7.4	10.3	8.4	9.3	14.6
9	1	0.58	5.4	24.6	8.4	17.2	14.4	4.5	16.5	3.4
10	−2.5	0.74	12.1	16.7	12.2	27.9	10.8	2.2	10.8	3.7
11	−2.5	0.20	9.1	16.1	4.1	15.4	−0.6	−2.9	1.0	−5.6
Mean	0.7	0.66	10.9	14.5	11.7	18.4	9.0	7.0	6.3	9.1
± SD	1.9	0.51	7.5	12.0	6.7	8.3	6.5	9.6	16.7	10.2

^a^change from baseline to post ^b^average change from baseline to training in Joules (−) Negative/Eccentric work (+) Positive/Concentric work

### Recovery outcomes

We evaluated how well the components of mechanical work during training could act as predictors of measures of patient recovery. Our primary clinical outcome included changes in UEFM from Baseline (session 2) to Post (session 8). Beyond standard clinical assessments, we also evaluated changes in motor exploration performance. We employed an engineering metric, previously described in [15], which captures the “maximum” range of movement velocities spanned during motor exploration. Velocity coverage is expressed as the estimated area of two dimensional velocity data (in units of m^2^/s^2^). To determine velocity coverage, we first calculated the 90th percentile speed within 64 equally spaced bins radially aligned from the zero-velocity center within the range of 0-2π. We then calculated the area within the boundary formed by connecting the points representing the 90th percentile speed within each bin. We considered the change in velocity coverage from Baseline (session 2) to Post (session 8) as an additional outcome prediction.

### Prediction model

We employed a Least Absolute Shrinkage and Selection Operator (LASSO) predictive model to predict recovery outcomes using our work features [27]. The LASSO method is a special case of regularized least squares regression which incorporates an additional penalty term on the L_1_ norm of the model coefficients. We chose LASSO because it has the advantage over alternative approaches of enhancing the interpretability of the results by reducing the number of features used by the model. We used a first-order LASSO model represented by the following formula in Lagrangian form that determines a set of fitted coefficients such that:

$$ {\hat{\beta}}_{lasso}=\underset{\beta }{\mathrm{argmin}}\left\{\frac{1}{2}\sum \limits_{i=1}^N{\left({y}_i-{\beta}_0-\sum \limits_{j=1}^J{x}_{ij}{\beta}_j\right)}^2+\lambda \sum \limits_{j=1}^J\left|{\beta}_j\right|\ \right\} $$

where N equals the number of experimental participants (22 in total), J equals the number of features (9 in total), y_i_ is the outcome measure, x_i_ = [X_i1_, …,X_ip,_] represents the eight components of work features and an additional categorical feature representing training group denoted X_ij,_ (i = 1, …,N, j = 1, …,J), β_j_ is the coefficient of the j-th feature and β_0_ is the intercept. The model features were standardized to account for relative differences in magnitude between the components of work. λ represents the non-negative penalty term that controls the degree of regularization by effectively driving the coefficients of features that are unhelpful to the predictions to zero. This results in a reduced feature set incorporated by the model. For our model predictions, we chose the largest λ value (λ_1SE_) where the cross-validated mean-square error MSE_CV_ (i.e. an estimation of how well the model would predict new data) is within one standard error of the minimum MSE_CV_ [27].

Our primary goal in using LASSO regression was to determine the most important predictors of recovery outcomes, particularly among the work features (See Ranking the Features). In addition, we used LASSO regression to perform an exhaustive analysis of the sensitivity in the predictive ability of our work features. We relied on the adjusted coefficient of determination (R^2^) as our primary metric which measures the proportion of variance in our recovery outcomes that can be explained by the work features selected by LASSO. We determined the sensitivity in R^2^ to different data splits using 5-fold cross-validation with 231 repeats. Each repeat uniquely split the participant data into the five different folds. For each repeat, we chose the LASSO model where λ = λ_1SE_, then trained the model using all the data and calculated the R^2^ of the resulting predictions. As a secondary metric we calculated the mean squared error (MSE) of the trained model predictions and compared to the MSE_CV_ in order to assess the degree of overfitting. Our repeated cross-validation method provides a robust estimate of the variability in R^2^ and MSE to different choices of λ. As an additional measure of sensitivity, we repeated the cross-validation procedure with six bootstrap datasets. We constructed each of these datasets by randomly resampling, with replacement, the original dataset (all 96 trials). Thus, our measure on the quality of our model predictions included 1617 R^2^ estimates. Our bootstrap method increases confidence in our R^2^ and MSE estimates.

### Ranking the features

Our primary objective in using LASSO regression was to determine the most important predictors of recovery outcomes, particularly among the work features. LASSO conveniently reduces the number of features used in the model; however, there are no built-in methods for ranking the model predictors based on importance. We rely on our previously described methods in which, as a first step, the ranking of features was based on the frequency each was selected in the model [28]. The selection frequency metric, expressed as a percentage, represents the number of times in total each feature is included in the model (i.e. assigned a non-zero coefficient) across the 231 5-fold cross-validation repeats for each bootstrap dataset. Thus, the maximum possible number of times a given feature could be selected was 1617. Next, we used a heuristic approach, which deviates slightly from our previous methods [28], to determine the important model features. Here, we evaluated model performance (R^2^) with each successive removal of a feature, rather than removing each feature individually, starting with the highest ranked feature (i.e. the most selected) and compared to the model predictions that included the full feature set (Full Model). By excluding features successively and in this order, we determine the extent to which the remaining features are able to compensate for the excluded features and where model performance starts to diminish when they can no longer compensate.

## Results

We investigated how practice energetics (mechanical work) performed by stroke survivors could explain differences in recovery outcomes. Our use of a multiple regression model (LASSO) revealed more accurate predictions using changes in velocity coverage (coefficient of determination: mean R^2^ ± SD; 0.36 ± 0.03), as compared to changes in our primary clinical outcome UEFM (− 0.0007 ± 0.005). Our analysis also revealed that ‘training group’ was the most frequently selected feature in each of these models. This result suggests a significant group effect contributed uncertainty to the model predictions, and more importantly, in determining the work features most important to recovery. Thus, in the following analysis, we performed separate predictions for each group.

### Energetics relate to outcomes

**Fig. 2:**
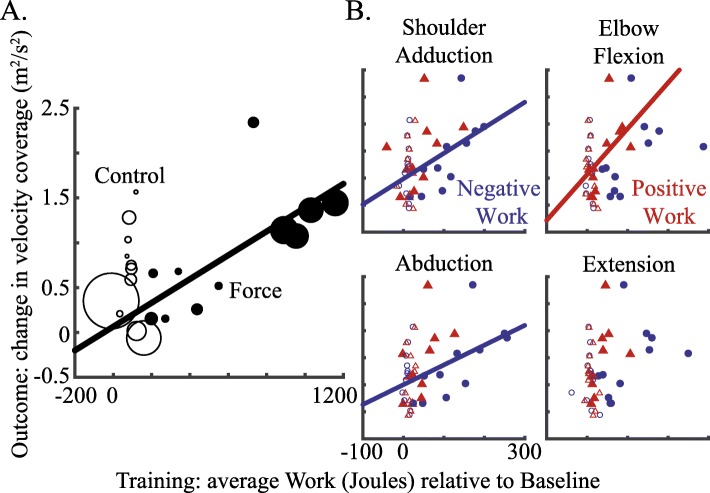
Correlation analysis. **a** The total mechanical work performed during motor exploration force training significantly correlated with changes in velocity coverage. Each data point represents an individual participant. The size of each data point is proportional to that participant’s velocity coverage during Baseline (session 2). For the Force group (black closed circles), participants with greater initial velocity coverage (evident of larger data points) tend to exert more total work during Training and showed greater gains in velocity coverage. This trend was not observed in the Control group (black open circles). **b** The breakdown of work reveals subcomponents that significantly correlated with changes in velocity coverage. A single pair of open (Control group) or closed (Force group) red triangle (Positive Work) and blue circle (Negative Work) along the x axis (the Training axis on each plot) represents an individual participant. Regression lines only shown for statistically significant correlation (α < 0.05) observed in the Force group

Prior to our main analysis, we inspected how training energetics relate to patient outcomes (Fig. 2). We first examine the degree that the total work performed (change from Baseline to Training in Joules, mean ± SE; Force, 617.7 ± 106.2; Control, 87.5 ± 13.6) correlated with changes in our main clinical outcome measure (change in UEFM scores from Baseline to Post, mean ± SE; Force, 1.1 ± 0.4; Control, 0.7 ± 0.6). Because these observed changes in UEFM scores would not be considered “clinically important” [29], it may not be surprising that we failed to detect a trend for both groups (Force, *r* = − 0.29, *p* = 0.4; Control, *r* = 0.1, *p* = 0.77). However, beyond clinical outcomes, we focused our investigation on whether training energetics relates to changes in velocity coverage (change from Baseline to Post; Force, 0.88 ± 0.20 m^2^/s^2^; Control, 0.66 ± 0.15 m^2^/s^2^). We expected that this measure would be more sensitive to any recovery resulting from motor exploration training (Fig. 2a). Interestingly, we found a significant correlation for patients who trained with forces (*r* = 0.7, *p* = 0.1), but not the Control group (*r* = − 0.003, *p* = 0.99). It is worth noting that the Force group performed much greater levels of work than the Control group due to the presentation of interactive forces.

Besides the basic link we found between work and recovery, we were interested in pinpointing the specific *components of work* that best predict recovery (Table 1). As a preliminary inspection, we correlated changes in work for each component with the recovery outcomes (Fig. 2b). For the Force group, we found that negative work during shoulder adduction (*r* = 0.73, *p* = 0.01) and abduction (*r* = 0.71, *p* = 0.01) and positive work during elbow flexion (*r* = 0.65, *p* = 0.03) significantly correlated with changes in velocity coverage, but not positive work during elbow extension (*r* = 0.54, *p* = 0.08), negative work during elbow flexion (*r* = 0.45, *p* = 0.17) and extension (*r* = 0.46, *p* = 0.15), or positive work during shoulder adduction (*r* = 0.45, *p* = 0.16) and abduction (*r* = 0.56, *p* = 0.08). We failed to detect any trends for the Control group. Our correlation analysis also did not reveal significant effects for changes in UEFM for both groups.

### Model performance

**Fig. 3:**
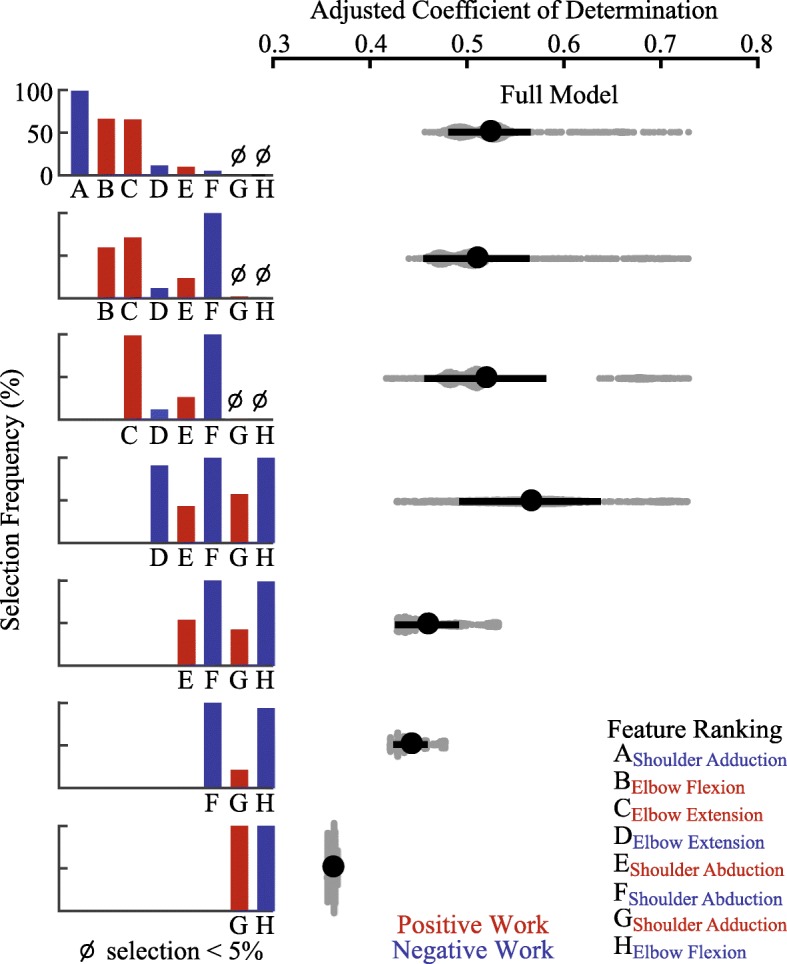
Model predictions and feature selection. Predictions of patient recovery, in terms of changes in velocity coverage, using multiple regression analysis. Each gray dot represents a single repeat of a cross-validation staggered for easy visualization by fitting a probability density function. Each black dot and bar represents the mean R^2^ ± SD. Positive (concentric) and negative (eccentric) work features are indicated in red and blue, respectively. The negative work in shoulder adduction and positive work in elbow flexion and extension features were selected most often by the LASSO model across the cross-validation repeats. The successive removal of the four most selected features resulted in a diminishing return of model accuracy. The full model equation is represented as y = [4.85A + 1.46B + 2.75C - 0.41D + 0.16E + 0.09F + 211.0] × 10^− 3^, where model coefficients assigned to each feature were averaged across cross-validation repeats

We performed a rigorous statistical analysis to determine how well components of work during training collectively predict patient outcomes using multiple regression analysis (Fig. 3). Unsurprisingly, we found better predictions for the Force group compared to the Control group. Our model predicted changes in velocity coverage with a coefficient of determination of 0.52 ± 0.043 (mean R^2^ ± SD) for the Force group (shown in Fig. 3, Full Model) and 0.34 ± 0.15 for the Control group. The mean squared error of the trained model predictions was 0.21 ± 0.02 (MSE ± SD) for the Force group and 0.17 ± 0.04 for the Control group. While our modeling approach provided a robust estimate of the predictive power of the components of work, we also wanted to evaluate how well the model might perform in predicting new data. We found the estimated predicted mean squared error in cross-validation was 0.64 ± 0.09 (MSE_CV_ ± SD) for the Force group and 0.46 ± 0.06 for the Control group. In contrast to changes in velocity coverage, prediction accuracy for changes in UEFM score was substantially lower for both groups (mean R^2^ ± SD; Force, 0.16 ± 0.14; Control, 0.25 ± 0.22).

### Feature importance

Beyond proposing an overall predictive model, we used LASSO to determine the relative importance of each work feature to predicting outcomes (Fig. 3). We first examined the selection frequency of each work feature by our LASSO model which determined the order of feature removal used in our subsequent analysis. Our analysis showed that negative work in shoulder adduction (98.9% selected), positive work in elbow flexion (66.1%) and extension (65.5%) were selected most. The remaining features were selected considerably less than the top features (negative work in elbow extension and positive and negative work in shoulder abduction were less than 12%, positive work in shoulder adduction was less than 5% and negative work in elbow flexion was not selected).

As a complement to selection frequency, we analyzed how removing features influenced the accuracy of model predictions. Interestingly, we found that removing the top feature (negative work in shoulder adduction) resulted in little change in model performance (mean R^2^ ± SD, 0.51 ± 0.06). However, this apparent lack of sensitivity corresponded to an increase in the selection of negative work in shoulder abduction. Similarly, removing elbow positive work, selection frequency for negative elbow work increased, resulting in modestly improved model performance (0.57 ± 0.07). The apparent replacement of model features that resulted in small changes in model performance indicates that these features are highly correlated and are able to compensate for any loss in variance explained. Model performance started to diminish after removing the fourth ranked feature (negative work in elbow extension, 0.46 ± 0.03) which indicates that the remaining features were unable to compensate for the first four features removed.

### PCA model

The results of our feature ranking suggested that several of the subcomponents of work are mutually correlated. To verify the dimensions required for predicting outcome, we performed an additional preprocessing step using principal component analysis (PCA) (See Supplemental Fig. 1). PCA effectively mapped the highly correlated features into an orthogonal space to obtain a set of uncorrelated principal components which were then used as candidate features to predict recovery (changes in velocity range). Our main observation was that prediction accuracy improved with each additional principal components included in the model. The highest R^2^ occurred using all eight possible principal components (mean R^2^ ± SD, 0.80 ± 0.05); however, this trend corresponded with increasing model uncertainty. The most reliable model, however, included the first four principal components (0.66 ± 0.02) since it corresponded with the largest change in R^2^ from the preceding three component model and a diminishing return of R^2^ on subsequent higher dimensional models. We obtained the coefficients of the four principal component model (See Supplemental Fig. 1) which revealed that negative work in shoulder adduction, positive work in the elbow, and negative work in elbow flexion contributed the most in terms of relative magnitude, similar to our previous results (compare to our full model in Fig. 3).

## Discussion

The goal of this investigation was to identify the subcomponents of mechanical work that were most associated with recovery. This study provided evidence that the degee of recovery depends on the amount of mechanical work performed while experiencing interactive forces during therapy. Our main finding was that negative work (eccentric) during horizontal shoulder adduction and positive work (concentric) during elbow flexion and extension were the most important features in predicting improvement, measured by range of movement velocities. These results imply that more energy exerted in braking (i.e. energy dissipation) shoulder motion and driving (i.e. energy input) elbow motion can heighten recovery.

Beyond identifying these important subcomponents of work, our results showed that the energetics of patient limb motion can account for 52% of the variance in the increases in measures of independent movement capabality among the Force group. Our findings highlight the importance of active involvement in recovery, and in particular, how components of energetics may be used to evaluate involvement during robotic training. The differences in predictive power that we found between the subcomponents of work suggest that certain forms of motor expression are important in recovery. Importantly, our results also demonstrate that robotic training forces can provoke increases in both positive (concentric) and negative (eccentric) work compared to the Control group that experienced equivalent amounts of repetitive practice without forces (See Fig. 2). Yet, according to our feature-selection procedure, specifically increasing negative work in the shoulder and positive work in the elbow led to corresponding increases in recovery.

Allowing patients to practice using a variety of movement patterns could have introduced several factors that contributed to increases in velocity coverage. Motor exploration differs from tasks patients typically perform in other robot training studies where the control mechanisms associated with upper limb recovery may have direct clinical interpretations [30, 31]. Our results show that the shoulder contributed the most to energetics overall which is likely due to its importance to workspace area [32]. However, the fact that negative work in the shoulder was the strongest predictor suggests that participants actively resisted the destabilizing force fields mostly by using the shoulder to dissipate energy. It is possible that such dissipation of energy in the shoulder must be coordinated with exertion of positive work (concentric) in the elbow [19, 20]. This result would be unsurprising since stroke survivors commonly exhibit abnormal flexion synergy during arm motion [33, 34]. While appropriate acceleration and deceleration is known to be fundamental in reaching [35], sustained movement in particular does not engage the motor system to use strategies for dissipating energy [9]. It is possible that negative work at the shoulder was the preferred mechanism to break out of abnormal synergies; thus, allowing for greater independence of elbow flexion and extension motion. Further analysis is needed to fully inspect how different types of work (negative/eccentric and positive/concentric work) are associated with the specific types of movement patterns patients made during exploration training.

Our model achieved 52% of variance explained even though the features considered were only components of work. However, it is important to note that our model predictions and identification of the important work subcomponents apply only to when robot forces were introduced. Unsurprisingly, our analysis showed that patients who train with the velocity-augmenting forces (Force group) exhibited substantially more work across training than the Control group. The fact that our prediction model identified the training group factor as an important feature suggests that the physical presence of robotic forces is necessary for predicting recovery from measures of involvement, consistent with previous studies [17]. It is interesting, however, that the Control group showed similar gains in velocity coverage (and UEFM scores) despite the absence of training forces. This could point to general therapeutic benefits of motor exploration training with simple feedback that encourages randomness. Alternatively, it is possible that motor exploration without forces reinforces stereotypical movement patterns in stroke, but at greater intensities. As our coverage metric only captures the overall change in patients’ ability to sustain greater velocities, additional analysis might elucidate whether the training groups differed in the particular forms of motor behaviors expressed during training that led to similar outcomes.

An important limitation of this study is that our key metric, velocity range, revealed improvements that might not be representative of general motor function. It is possible that the observed changes simply reflect a shift in motion patterns in response to training conditions that represent some combination of long-term adaptation or short-term changes in reward-motivated behavior. This may be one reason why we did not find a relationship between energetics and clinical outcomes. Because power is governed partly by speed, and work is the integral of power, it may be of little surprise that work is related to velocity coverage. Changes in UEFM scores may have been too small to be considered clinically relevant. Each participant’s customized force field was designed to push their hand towards underrepresented velocities, which were nearly always higher. Velocities in the arm observed in this study might not transfer to other forms of motor function. Nevertheless, we previously provided evidence [15] that increases in velocity coverage corresponded with faster task execution in “transfer” tasks that were not trained, both important hallmarks of functional gain.

Our PCA analysis highlights a limitation of multiple regression, and feature selection using LASSO. We entertained alternative strategies to deal with the multicollinearity problem; including, re-ranking the features after removing a covariate and removing likely pairs of covariates in random order. The predictive power of our model improved substantially with the use of PCA, which suggests that the highly correlated metrics introduced a source of noise or unnecessary model complexity. Our results also suggest that a minimum of four dimensions (the first four principal components of the work features) are required in our prediction model of recovery, consistent with the results from our feature removal procedure. It is important to note that, in contrast to our model that excluded covariate features, the PCA-LASSO model relies on the independent contributions of each work feature (See Supplemental Fig. 1D). This indicates that each individual component of work could play an important role in model predictions when the shared variance is removed with PCA. It is likely that the higher dimensional principal components describe additional noise within the work features, thus resulting in model uncertainty. Machine learning methods have been used recently to not only identify relationships but to also understand levels of uncertainty using bootstrapping and cross-validation.

While the focus of this investigation was to understand the outcome relation with mechanical work, additional factors can and should be considered to further enhance prediction of recovery. Individual differences in patient involvement might be described in a variety of ways, for example, movement frequency [36], muscle activations [37], end-point force production [18], or even considering the energetics of the robot [38]. It is also likely that initial patient ability dictates their capacity to do work. In fact, we found trends between the initial characterization of velocity coverage during motor exploration and changes due to training (See Fig. 2). This is consistent with previous studies that have found that certain features of movement capabilities at the beginning of training can predict changes in clinical outcomes [24], for example, simple measures such as movement speed [39]. Further analysis is needed to determine the key factors, both in terms of patient characteristics and training conditions, that impact recovery from robot-assisted therapy. Another shortcoming may be that this model does not consider interaction terms between covariates. We believed that simple linear terms would serve as the best proof of concept, and LASSO provided quality feature selection abilities important for predicting recovery. Adding interaction terms might complicate the model, making it more difficult to interpret. Further work is needed to examine how such interaction effects can build upon the main predictive trends found in the current study.

Our implementation of an upper limb inverse dynamic model provides a convenient method for quantifying the energetic contributions of patients to movement. It is worth noting, however, that mechanical work cannot account for physiological preferences established by muscle length-force relationships, joint pain, co-contraction, or other differences that influence force or motion capacities. The interactive forces provided by the robot during training varied as a function of the patients’ instantaneous movement velocities without consideration of the differences in effort requirements at different joint angles. For example, it is possible that the forces provided too little assistance at the extremes of motion. Researchers have recommended alternative “weighted” measures of work to account for these important physiological relationships [40] which also require thoughtful consideration for future iterations of force field design.

Last, it is important to clarify that the LASSO methods we present in this paper do not substitute true experimental validation. In fact, our results show that the cross-validated mean squared error (i.e. the estimated error in predicting future data) was greater than the mean squared error of the trained model predictions which indicates some degree of model overfitting. Our overall approach of applying exhaustive measures (repeated cross-validation and bootstrapping), and subsequent feature removal analysis based on the selection frequency metric, offered a process for identifying the important model features given our limited data set. While LASSO is known to enhance predictive quality and prevent overfitting, it provided a convenient method for examining feature selection in our analysis. Future analysis should consider alternative modelling techniques, including other regularization methods, to determine whether LASSO does in fact improve predictive quality.

## Conclusions

This study examined the special case of training with interactive forces and provided evidence that the degree of recovery in stroke survivors depends on the amount of mechanical work performed. In addition, our analysis revealed that the components of work most important to predicting recovery were those associated with eccentric shoulder and concentric elbow motions. We remain cautious about how our predictions might generalize, especially with respect to other types of training (e.g. reaching) that involve different forms of force interactions. However, our results highlight the importance of quantifying patient involvement, as well as revealing how specific forms of involvement should be targeted. Our findings demonstrate the potential for energetic measures in the evaluation and design of robot assisted therapy.

## Supplementary information

**Additional file 1: Figure S1.** PCA model predictions. Predicting patient recovery (changes in velocity coverage) due to robotic force training using features obtained from the principal components of the work subcomponents. **a** The successive inclusion of features increased average accuracy of prediction, but with decreased certainty (each gray dot represents a single cross-validation repeat, black dot and bar represents the mean R^2^ ± SD). **b** The loadings determined by PCA for each individual principal component assigned to the work subcomponents. The first three principal components appeared to correspond to differences between 1) low and high magnitude, 2) shoulder and elbow, 3) positive (concentric) and negative (eccentric) forms of work. **c** The coefficients the LASSO model assigned to each principal component feature (black dot and bar represents the mean ± SD calculated across cross-validation repeats). **d** The combined weights representing the relative contribution of each work subcomponent to model predictions as principal component features were successively included. The PCA model derived using the first four principal components closely resembles the full model in Fig. 3, which suggests a greater predictive importance of negative work in the shoulder adduction and positive work in the elbow.

## Data Availability

Portions of the datasets used and/or analyzed for this study were previously published (DOI: 10.1109/TNSRE.2017.2763458). Additional datasets generated for the purpose of the analysis presented in this study are available from the corresponding author on reasonable request.

## Abbreviations

LASSO: Least Absolute Shrinkage and Selection Operator
m: Meters
PCA: Principal component analysis
r: Correlation coefficient
R^2^: Coefficient of Determination
s: Seconds
SD: Standard deviation
UEFM: Upper extremity Fugl-Meyer
